# Relationships Between Mathematics Performance and Attitude to Mathematics: Influences of Gender, Test Anxiety, and Working Memory

**DOI:** 10.3389/fpsyg.2022.814992

**Published:** 2022-03-07

**Authors:** Ann Dowker, Hannah Sheridan

**Affiliations:** ^1^Department of Experimental Psychology, University of Oxford, Oxford, United Kingdom; ^2^Lady Margaret Hall, University of Oxford, Oxford, United Kingdom

**Keywords:** mathematical performance, attitudes to mathematics, test anxiety, working memory, gender differences, adults

## Abstract

Many studies have indicated that mathematics anxiety, and other negative attitudes and emotions toward mathematics, are pervasive and are associated with lower mathematical performance. Some previous research has suggested that working memory is related to both mathematics anxiety and mathematics. Moreover, both gender and chosen course of study (sciences vs. humanities) appeared likely to influence students’ attitudes to mathematics. In the present study, 40 university undergraduates completed a battery of assessments investigating working memory, attitude to mathematics, test anxiety. and mental and written arithmetic. Attitudes to mathematics were significantly associated with the other variables: working memory, test anxiety, and both measures of mathematical performance. The other variables were not strongly associated with one another. There were no gender differences in mathematical performance, but females exhibited more negative attitudes to mathematics and higher test anxiety than males. After controlling for test anxiety, there ceased to be significant gender differences in attitudes to mathematics. Science students had more positive attitudes to mathematics than humanities students, but the groups did not differ in test anxiety, Science students were better at written but not mental arithmetic. They were also better at working memory, but this was not a significant covariate when the groups were compared on mathematical performance and attitudes to mathematics The results are discussed, with particular focus on implications for future research on influences on mathematics anxiety.

## Introduction

Numerous studies indicate that attitudes to mathematics are often highly negative, ranging from boredom to severe fear and anxiety. Mathematics anxiety has been defined as “feelings of tension or anxiety that interfere with the manipulating of numbers or the solving of mathematical problems” (Richardson and Suinn, 1972). Estimates of the frequency of mathematics anxiety range from 11% (Betz, 1978) to 68% (Richardson and Suinn, 1972). The frequency of mathematics anxiety will depend both on the nature of the sample and on how “mathematics anxiety” is defined, but even the lower estimates suggest that it is significant problem for many. Moreover, even people who do not have mathematics anxiety as such may have negative attitudes to mathematics and regard it as boring, a waste of time, too difficult for them, and/or irrelevant to their own lives (see Fennema and Sherman, 1976).

Many studies have also found that mathematics anxiety and other negative attitudes to mathematics are associated correlationally, with poorer performance in mathematics (e.g., Hembree, 1990; Ma and Kishor, 1997; Maloney et al., 2011; Carey et al., 2016; Dowker et al., 2016; Zhang et al., 2019; Abín et al., 2020).

This does not mean that the direction of causation is always from anxiety or negative attitudes to performance. Weaknesses in mathematics may cause failures and other negative experiences, which then lead to anxiety and other negative attitudes (Núñez-Peña and Suárez-Pellicioni, 2014). It is generally thought now that there is a bidirectional relationship between attitudes and performance. For example, Maloney and Beilock (2012) propose that a combination of social factors and pre-existing mathematical difficulties results a negative attitude to mathematics. This in turn impedes subsequent performance in mathematics, resulting in a vicious circle.

This study also investigates; the possible relationship between attitude to mathematics and working memory. There are some studies that suggest that mathematics anxiety may impair performance by overloading working memory (Eysenck and Calvo, 1992; Ashcraft and Kirk, 2001). Beilock and DeCaro (2007) found that, in studies of mathematics anxiety, there was only a correlation between mathematics anxiety and mathematics performance when the task given required significant use of working memory resources. Ashcraft and Kirk (2001) supported this idea by finding that people with higher mathematics anxiety demonstrated lower working memory than people with less mathematics anxiety, particularly in tasks involving calculation. Caviola et al. (2012) suggested, similarly, that if anxiety affects working memory, it should have an especially strong effect on arithmetic, as mathematics requires working memory. Vukovic et al. (2013) drew together these ideas and showed that, in a longitudinal study of 113 children, the relationship between mathematics anxiety and performance is greater in those with poorer working memory abilities. This study will examine the relationships between working memory, mathematics anxiety, and mathematical performance in adults.

Some apparent “mathematics anxiety” may in fact reflect a less specific anxiety about academic subjects and especially about tests and examinations. There is usually found to be a high correlation between mathematics anxiety and test anxiety, with typical correlations ranging from 0.3 to 0.5 (Hembree, 1990; Ashcraft et al., 1998). However, mathematics anxiety is not *just* a form of test anxiety, studies generally show a higher correlation between different measures of mathematics anxiety (0.5–0.8) than between mathematics anxiety and test anxiety or general anxiety (Dew et al., 1983; Hembree, 1990; Ashcraft and Ridley, 2005).

A number of studies have attempted to investigate and disentangle the interrelationships between mathematics performance, mathematics anxiety, working memory, and sometimes other characteristics. These studies have given interesting but sometimes somewhat conflicting results. For example, structural equation modeling has been used to obtain a finer-grained analysis of the relationships between mathematical performance, mathematics anxiety, working memory, and other cognitive skills, sometimes producing somewhat contrasting results. Skagerlund et al. (2019) used structural equation modeling to analyze the interrelationships between mathematics anxiety, mathematical performance, and working memory. They found three separate pathways from mathematics anxiety to mathematical performance: a direct effect of mathematics anxiety on performance; an indirect effect *via* effects on symbolic number processing; and an indirect effect *via* effects on working memory. Douglas and Lefevre (2018) also used structural equation modeling to investigate the interrelationships between the above variables and the mathematics-related skills of quantity processing and spatial processing. Although all these variables were correlated, no direct link was found between mathematics anxiety and either quantity processing, spatial processing or working memory; nor were the relationships between the latter abilities and mathematics performance indirectly affected by working memory.

Meta-analyses have been used to combine the results of numerous studies in order to obtain more precise and detailed information about the relationships between mathematics anxiety and mathematical performance and factors that may contribute to such relationships. Zhang et al. (2019) and Barroso et al. (2021) carried out meta-analyses, both of which showed a moderate consistent negative correlation between mathematics anxiety and mathematical performance. The relationship was strongest in secondary school pupils and lowest in children in grades 3 to 5 and in college students. Zhang et al. (2019) also examined the effects of other demographic and methodological variables and found that the relationship between mathematics anxiety and performance was stronger for Asian than European students and was strongest among studies that used a custom test and studies that assessed problem-solving skills. A meta-analysis by Caviola et al. (2021) indicated that both mathematics anxiety and test anxiety were negatively associated with mathematical performance. Working memory had a weak moderating effect on these relationships. Namkung et al. (2019) carried out a meta-analysis specifically of studies of school age pupils and found that the relationship between mathematics anxiety and performance was strongest when the mathematics anxiety measures included both affective and cognitive components; when the mathematics performance measures involved formal assessments that influenced or reflected school grades; and when the mathematics performance measures involved advanced and/or multi-step arithmetic.

One consistent finding from most previous studies is that there are significant gender differences in attitudes to mathematics. Most studies indicate that females show higher mathematics anxiety than males (e.g., Hembree, 1990; Miller and Bichsel, 2004; Devine et al., 2012; Ganley and Vasilyeva, 2014; Sarfo et al., 2020; Wang, 2020; Xie et al., 2020; Delage et al., 2021). Some studies have shown such gender differences even in children in the early years of primary school (Szczygiel, 2020), though many studies have not found such a difference (e.g., Harari et al., 2013; Ching, 2017; Mononen et al., 2021). Most of the studies (with a few exceptions) do not show gender differences in mathematical *performance*. Spelke (2005) reported that in countries where girls have equal education and opportunities, there is no significant gender difference in mathematics performance. Some studies suggest that mathematics anxiety has different effects on performance in males and in females, but the studies give conflicting results as to the direction of the gender difference. Hembree (1990) and Miller and Bichsel (2004) found that mathematics anxiety affected performance more in males than in females. Devine et al. (2012) found on the other hand that after controlling for test anxiety, mathematics anxiety had an independent effect on mathematics performance in girls but not in boys. Hembree (1990) drew attention to the lack of conclusive agreement across studies, as to relationship between gender, mathematics anxiety, and mathematical performance, which Birgin et al. (2010) later attributed to the lack of consistent measurement of mathematics anxiety. The current study, therefore, intends to further investigate the influence of gender on mathematics performance and on a measure related to mathematics anxiety while controlling for test anxiety.

The current study also intends to investigate the relationship between attitude to mathematics and degree subject of study. Betz (1978) found that correlations between mathematics anxiety and performance in university students differed according to course as well as gender. Ashcraft (2002) suggested that correlations between mathematics anxiety and performance could be because those that have higher levels of mathematics anxiety avoid situations involving mathematics, which may mean avoiding certain areas of study, and, thus, gain less practice in mathematics. Thus, the current study intends to investigate how gender and subject of study interact with any relationships between mathematics performance and mathematics anxiety. We tentatively propose that science students may have higher working memory than humanities students, because their area of study may require more short-term mental mathematical and logical calculations, as compared with analyses of long-term information. Popescu et al. (2019), found that mathematics graduate students scored higher on backward digit span and on another working memory task (forward letter span) than humanities graduate students.

The current study investigates relationships between all these variables; attitudes to mathematics, mathematics performance, gender, degree subject, and working memory. The participants in the study were Oxford University students and therefore could be assumed to exclude those with extremely poor mathematical performance (entry requirements usually include a high grade in mathematics at GCSE or equivalent). Therefore, it was decided to use a mathematics attitude measure that did not focus solely on negative attitudes, but included both enjoyment and anxiety. The measure chosen was Aiken’s (1974) Mathematics Enjoyment Scale. This also had the advantage of not being very time-consuming, though this also comes with the disadvantage of not being able to include several different factors. Because many of the questions are in fact about anxiety, the construct measured will be termed Mathematics Enjoyment/Anxiety.

Given previous findings about the complicated relationships between mathematics anxiety, test anxiety, and mathematics performances anxiety, mathematics performance (e.g., Devine et al., 2012), a standard measure of test anxiety was also included. The measures of mathematics performance were chosen because they both included only topics that are covered in compulsory school mathematics courses. Thus, it is unlikely that the specific content learnt in the degree would be an additional factor influencing mathematics performance. The decision to use a numerical working memory test seemed most appropriate to the field of mathematics anxiety as this tests working memory for numbers, which is required in mathematics.

Our predictions were (1) that mathematics performance would correlate with both attitudes to mathematics and working memory; (2) that both mathematics anxiety and working memory would be independent predictors of mathematics performance in a multiple regression; (3) that general test anxiety would correlate with both mathematics performance and attitudes to mathematics; (4) that mathematics performance measures and test anxiety would be independent predictors of mathematics anxiety; (5) that females would show more mathematics anxiety and more test anxiety than males; (6) that males and females would not differ in actual mathematical performance or in working memory; (7) that gender differences in mathematical performance would reduce after controlling for test anxiety; (8) that science students would perform better than humanities students on mathematics measures; (9) that science students would show higher mathematics anxiety than humanities students; (10) that science students would show higher working memory than humanities students; and (11) that differences between science and humanities students would reduce after controlling for working memory.

## Materials and Methods

### Design

A between-participants design was used. The grouping factors were gender (male vs. female) and subject of study (sciences vs. humanities). There were five dependent variables: two different mathematics test scores, digit span, mathematics anxiety, and test anxiety. Participants were selected to ensure equal numbers of participants falling into each of the grouping categories.

### Participants

Participants were 40 University Undergraduates aged 18–25. Ten were males studying sciences, 10 females studying sciences, 10 males studying humanities, and 10 females studying humanities. University subjects were classed as sciences or humanities on the basis of the division in which the university classed them: sciences if classed in the Medical Sciences division or Mathematical, Physical and Life Sciences division; and humanities if classed in the Humanities division or Social Sciences division.

Participants were recruited through advertisement *via* email and social media and through social contacts and word-of-mouth. They were given an information sheet about the tasks that they would be given and then signed a consent form.

Ethical approval was sought and granted by the Central University Research Ethics Committee of Oxford University.

### Tasks

#### Attitude Measures

1. A measure of Test Anxiety. The measure used was Sarason’s (1977) Test Anxiety Scale. This had been shown to have test–retest reliability scores in the 0.80 (Zeidner and Matthews, 2003). It has also been found to correlate well with other test anxiety scales, indicating good concurrent validity. In the present study, Cronbach’s alpha for this measure was 0.91.
2. A measure of attitudes to mathematics. The measure used was Aiken’s (1974) Mathematics Enjoyment scale. This test had a Cronbach alpha of 0.95 in Aiken’s (1974) original study; 0.88 in Watson’s (1983) validation; and 0.87 in the sample tested in the present study. Both Aiken (1974) and Watson’s (1983) obtained highly significant correlations with a range of measures of mathematical performance and attitudes to mathematics, indicating good concurrent validity.

#### Working Memory Test

3. WAIS Digit Span subtest. This was taken from the Wechsler Adult Intelligence Scale-IV (Wechsler, 2008). This involves repeating strings of numbers forward and backward. For the purpose of the present study, the Backward Digit Span was used, as this is a purer measure of *working memory*.

#### Mathematics Tests

4. WAIS Arithmetic subtest (Wechsler, 2008). This was taken from the Wechsler Adult Intelligence Scale-III. It is an orally presented test of word problem-solving with an oral response. The scaled score was the measure used in the analysis. Cronbach’s alpha was 0.9 both in the original standardization and in the present study.
5. Test 2 of Hitch’s (1978a) Numerical Abilities Tests. This was a written test, involving mathematical questions on fractions, decimals, percentages, and arithmetic functions. Participants were given up to 20 min to complete this without a calculator. In the original study, the split-half reliability computed by the Spearman–Brown formula was 0.97. In the present study, Cronbach’s alpha was 0.95.

### Procedure

Participants were asked to read an information sheet and sign a consent form and were then told the tasks they were going to complete. Participants were given these tasks in a quiet room with only the researcher present. They were first given the Backward Digit Span and WAIS Arithmetic test (Wechsler, 2008). They were then given the Test Anxiety scale and then the Mathematics Enjoyment Scale, untimed. These were presented on paper, and participants were asked to complete them by hand. Finally, participants were given the Written mathematics test. The decision was made to put the Written mathematics test last, so that self-perceived performance on it would not impact responses given on the attitude measures.

### Analysis

IBM SPSS Version 25 was used to analyze the data (SPSS, IBM, 2017).

## Results

Scaled scores were coded for Arithmetic using the scoring guide in the WAIS scoring manual (Wechsler, 2008). Since only Backward Digit Span and not Forward Digit Span was included in the analyses for this study, no scaled score was coded for Digit Span.

Arithmetic raw scores ranged from 12 to 21 (*M* = 17.93, *SD* = 1.94) and scaled scores from 10 to 17 (*M* = 8.45, *SD* = 2.3). Written mathematics test scores ranged from 19/40 to 40/40 (*M* = 33.25, *SD* = 6.03). Backward Digit Span scores ranged from 5 to 13 (*M* = 20.30, *SD* = 3.94). Test anxiety scores ranged from 4 to 27 (*M* = 15.15, *SD* = 5.13). Mathematics Anxiety/Enjoyment scores ranged from 12 to 48 (*M* = 25.58, *SD* = 11.53).

### Pearson’s Correlations

Pearson’s correlations were examined between Arithmetic scaled score, Written Mathematics test score, Backward Digit Span, Test Anxiety, and Mathematics Anxiety/Enjoyment. These correlations are shown in Table 1. The correlation between Arithmetic scaled score and Written mathematics test score did not reach significance (*p* = 0.07). Backward Digit Span correlated significantly with Arithmetic scaled score, but not with Written mathematics test score. Mathematics Anxiety/Enjoyment correlated significantly with all the other variables: positively with test anxiety and negatively with backward digit span, arithmetic scaled score and written Mathematics test score. Test anxiety only correlated with Mathematics Anxiety/Enjoyment.

**Table 1:** Pearson’s correlations for arithmetic scaled score, written mathematics test score, backward digit span, test anxiety, and mathematics anxiety/enjoyment.

	Arithmetic Scaled Score	Written mathematics	Backward Digit Span	Test Anxiety	Math. Anxiety/Enjoyment
Arithmetic Scaled Score	–	0.26 (*p* = 0.1)	0.3 (*p* = 0.06)	0.09 (*p* = 0.46)	−0.379^*^ (*p* = 0.016)
Written Mathematics	–	–	0.27 (*p* = 0.1)	−0.12 (*p* = 0.47)	−0.344^*^ (*p* = 0.03)
Backward Digit Span	–	–	–	0.163 (*p* = 0.316)	−0.37^*^ (*p* = 0.018)
Test Anxiety	–	–	–	–	0.423^**^ (*p* = 0.007)

* *p* < 0.05;

** *p* < 0.01.

### Multiple Regressions

An entry-type multiple regression was carried out with Arithmetic scaled score as the dependent variables, and Backward Digit Span, Test Anxiety and Mathematics Anxiety/Enjoyment as the predictors. *R*^2^ was 0.17. The model did not explain a significant amount of the variance [*F*(3,36) = 2,5; *p* = 0.07]. None of the individual predictors was significant for Arithmetic scaled score: Neither Backward Digit Span, *β* = 0.22, *t*(3, 37) = 1.26, *p* = 0.217; Test Anxiety, *β* = −0.038, *t*(3,37) = −1.69, *p* = 0.834, nor Mathematics Anxiety/Enjoyment, *β* = −0.28, *t*(3,37) = −1.45, *p* = 0.15 proved significant.

Another entry-type multiple regression was carried out with Written mathematics test score as the dependent variable, and Backward Digit Span, Test Anxiety and Mathematics Anxiety/Enjoyment as the predictors. *R*^2^ was 0.14. The model did not explain a significant amount of the variance [*F*(3,36) = 1,97; *p* = 0.135]. None of the individual predictors was significant for Written mathematics test score: Neither Backward Digit Span, *β* = 0.17, *t*(3, 37) = 0.95, *p* = 0.347; Test Anxiety, *β* = −0.33, *t*(3,37) = −0.18, *p* = 0.838, nor Mathematics Anxiety/Enjoyment, *β* = −0.266, *t*(3,37) = −136, *p* = 0.18 proved significant.

Another entry-type multiple regression was carried out with Mathematics Anxiety/Enjoyment score as the dependent variable, and Backward Digit Span, Test anxiety and Arithmetic scaled score as the predictors. *R*^2^ was 0.42. The model explained a highly significant amount of the variance [*F*(3,36) = 8,81; *p* < 0.001]. Backward Digit Span was a highly significant predictor *β* = −0.385, *t*(3, 37) = − 2.79, *p* = 0.008; as was Test Anxiety, *β* = 0.462, *t*(3,37) = 3.51; *p* = 0.001. Arithmetic scaled score was not a significant predictor, *β* = 0.199, *t*(3,37) = −1.45; *p* = 0.156.

Another entry-type multiple regression was carried out with Mathematics Anxiety/Enjoyment score as the dependent variable, and Backward Digit Span, Test anxiety and Written mathematics test score as the predictors. *R*^2^ was 0.41. The model explained a highly significant amount of the variance [*F*(3,36) = 8,32; *p* < 0.001]. Backward Digit Span was a highly significant predictor *β* = − 0.4, *t*(3, 37) = − 2.95, *p* = 0.006; as was Test Anxiety, *β* = 0.466, *t*(3,37) = 3.55; *p* = 0.001. Written mathematics test score was not a significant predictor, *β = −*0.18, *t*(3,37) = 1.36; *p* = 0.183.

### Analyses of Variance

A two-factor between-participants Analysis of Variance was then conducted, with Gender (Male vs. Female) and Subject of Study (Science vs. Humanities) as the grouping factors and Arithmetic scaled score, Written mathematics test score. Backward Digit Span, Test Anxiety, and Mathematics Anxiety/Enjoyment as the dependent variables.

As Table 2 indicates, there were significant gender differences in both Test Anxiety and Mathematics Anxiety: females scored higher on both. There were no significant gender differences in either mathematical performance measure or on Backward Digit Span. There were significant course differences, with moderate effect size, for Written Mathematics (science students did better) and Mathematics Anxiety (humanities students scored higher), and one with lower effect size for Backward Digit Span (science students had longer spans). There were no significant interactions between course and gender. However, it should be noted that although Table 3 supports the lack of interaction between course and gender regarding the mean Mathematics Anxiety/Enjoyment scores, the standard deviation was much lower for male science students than for female science students or for humanities students of either gender.

**Table 2:** Results of analysis of variance with gender and course as grouping factors and arithmetic scaled score, written mathematics test score, backward digit span.

Source	Dependent Variable	df	Mean Square	*F*	Sig.	Partial Eta Squared
Gender	Arithmetic Scaled Score	(1,36)	0.625	0.182	0.672	0.005
	Written Mathematics	(1,36)	6.400	0.217	0.644	0.006
	Backward Digit Span	(1,36)	0.100	0.020	0.889	0.001
	Test Anxiety	(1,36)	108.900	4.477	0.041	0.111
	Mathematics Anxiety/Enjoyment	(1,36)	632.025	6.722	0.014	0.157
Course	Arithmetic Scaled Score	(1,36)	3.025	0.883	0.354	0.024
	Written Mathematics	(1,36)	348.100	11.807	0.002	0.247
	Backward Digit Span	(1,36)	22.500	4.480	0.041	0.111
	Test Anxiety	(1,36)	0.100	0.004	0.949	0.000
	Mathematics Anxiety/Enjoyment	(1,36)	950.625	10.110	0.003	0.219
Gender * Course	Arithmetic Scaled Score	(1,36)	0.025	0.007	0.932	0.000
	Written Mathematics	(1,36)	1.600	0.054	0.817	0.002
	Backward Digit Span	(1,36)	2.500	0.498	0.485	0.014
	Test Anxiety	(1,36)	2.500	0.103	0.750	0.003
	Mathematics Anxiety/Enjoyment	(1,36)	216.225	2.300	0.138	0.060

*Test anxiety and mathematics anxiety/enjoyment as the dependent variables*.

**Table 3:** Scores by gender and course for arithmetic (scaled score), written mathematics, backward digit span, test anxiety, and mathematics anxiety/enjoyment.

	Gender	Course	Mean	Std. Deviation	N
Arithmetic Scaled Score	Male	Science	14.4000	1.26491	10
		Hum.	13.8000	2.25093	10
		Total	14.1000	1.80351	20
	Female	Science	14.1000	1.66333	10
		Hum.	13.6000	2.06559	10
		Total	13.8500	1.84320	20
	Total	Science	14.2500	1.44641	20
		Hum.	13.7000	2.10513	20
		Total	13.9750	1.80438	40
Written Mathematics	Male	Science	36.80	3.521	10
		Hum.	30.50	6.721	10
		Total	33.65	6.141	20
	Female	Science	35.60	4.502	10
		Hum.	30.10	6.332	10
		Total	32.85	6.046	20
	Total	Science	36.20	3.982	20
		Hum.	30.30	6.359	20
		Total	33.25	6.029	40
Backward Digit Span	Male	Science	8.90	1.524	10
		Hum.	7.90	2.514	10
		Total	8.40	2.088	20
	Female	Science	9.50	2.121	10
		Hum.	7.50	2.635	10
		Total	8.50	2.544	20
	Total	Science	9.20	1.824	20
		Hum.	7.70	2.515	20
		Total	8.45	2.298	40
Test Anxiety	Male	Science	13.20	3.706	10
		Hum.	13.80	4.290	10
		Total	13.50	3.914	20
	Female	Science	17.00	6.515	10
		Hum.	16.60	4.766	10
		Total	16.80	5.559	20
	Total	Science	15.10	5.515	20
		Hum.	15.20	4.641	20
		Total	15.15	5.031	40
Mathematics Anxiety/Enjoyment	Male	Science	14.4000	1.89737	10
		Hum.	28.8000	11.24278	10
		Total	21.6000	10.77717	20
	Female	Science	27.0000	10.77033	10
		Hum.	32.1000	11.40614	10
		Total	29.5500	11.10938	20
	Total	Science	20.7000	9.92127	20
		Hum.	30.4500	11.15194	20
		Total	25.5750	11.52898	40

In order to investigate whether Test Anxiety was driving the results for gender differences and similarities, a one-way ANOVA was carried out, with Gender as the grouping factor, Test Anxiety as a covariate, and Arithmetic scaled score, Written mathematics test score, Backward Digit Span, and Mathematics Anxiety/Enjoyment as the dependent variables. Test Anxiety proved to be a significant covariate for Mathematics Anxiety/Enjoyment, *F*(1, 37) = 5.07, *p* = 0.03, $\eta_{p}^{2}$ = 0.12. There were now no significant gender differences in any variable, including Mathematics Anxiety/Enjoyment.

In order to investigate whether working memory was driving the results for course differences, a similar one-way ANOVA was carried out, with Course as the grouping factor, Backward Digit Span as a covariate, and Arithmetic scaled score, Written mathematics test score, Test Anxiety, and Mathematics Anxiety. Backward Digit Span was not a significant covariate for any of the dependent variables. Course differences continued to be significant for Written mathematics test score, *F*(1, 37) = 5.07, *p* = 0.004, $\eta_{p}^{2}$ = 0.201 and for Mathematics Anxiety/Enjoyment, *F*(1, 37) = 5.11, *p* = 03, $\eta_{p}^{2}$ = 0.121.

As the ANOVAs may have been somewhat underpowered due to the small sample size, they were supplemented with Bayesian analyses. Table 4 shows a Bayesian independent samples test for gender comparisons for Arithmetic scaled score, Written mathematics test score, Backward Digit Span, Test Anxiety, and Mathematics Anxiety/Enjoyment.

**Table 4:** Bayes factor independent sample test for differences between male and females (method = rouder)^a^.

	Mean Difference	Pooled Std. Error Difference	Bayes Factor^b^	*t*	df	Sig.(2-tailed)
Arithmetic Scaled Score	−0.2500	0.57663	3.962	−0.434	38	0.667
Written Mathematics	−0.80	1.927	3.990	−0.415	38	0.680
Backward Digit Span	0.10	0.736	4.269	0.136	38	0.893
Test Anxiety	3.30	1.520	0.596	2.171	38	0.036
Mathematics Anxiety/Enjoyment	7.9500	3.46097	0.476	2.297	38	0.027

a *Assumes unequal variance between groups*.

b *Bayes factor: Null vs. alternative hypothesis*.

As can be seen, the Bayes factor was high, favoring the null hypothesis, for the mathematical performance measures and Backward Digit Span, but much lower, giving greater support to the alternative hypothesis, for both anxiety measures.

Table 5 shows a Bayesian independent samples test for course comparisons for Arithmetic scaled score, Written mathematics test score, Backward Digit Span, Test Anxiety, and Mathematics Anxiety/Enjoyment.

**Table 5:** Bayes factor independent sample test for differences between science and humanities students (Method = Rouder)^a^.

	Mean Difference	Pooled Std. Error Difference	Bayes Factor^b^	*t*	df	Sig. (2-tailed)
Arithmetic Scaled Score	−0.5500	0.57113	2.870	−0.963	38	0.342
Written Mathematics	−5.90	1.678	0.035	−3.517	38	0.001
Backward Digit Span	−1.50	0.695	0.608	−2.159	38	0.037
Test Anxiety	0.10	1.612	4.297	0.062	38	0.951
Mathematics Anxiety/Enjoyment	9.7500	3.33764	0.137	2.921	38	0.006

a *Assumes unequal variance between groups*.

b *Bayes factor: Null vs. alternative hypothesis*.

As can be seen, the Bayes factor was high, favoring the null hypothesis, for Arithmetic Scaled Score and Test Anxiety, but much lower, giving greater support to the alternative hypothesis, for Written Mathematics, Backward Digit Span, and Mathematics Anxiety/Enjoyment. Thus, the results of the Bayesian analyses are concordant with those of the Analyses of Variance.

## Discussion

The results of this study confirm our first hypothesis and support many previous studies (Hembree, 1990; Ma and Kishor, 1997; Carey et al., 2016; Dulaney et al., 2017; Skagerlund et al., 2019; Zhang et al., 2019; Abín et al., 2020; Barroso et al., 2021; Caviola et al., 2021) in suggesting that there are some significant relationships between attitudes and performance. A mathematics anxiety and enjoyment measure correlated significantly with two different measures of mathematics: the WAIS Arithmetic subtest (Wechsler, 2008), which mainly tested oral arithmetic problem-solving involving relatively simple calculations and Hitch’s (1978a) Numerical Abilities Test 2, which mainly tested written, more complex calculations, and the understanding of fractions and percentages. The fact that it correlated with both simpler and more complex calculations suggests that its effect may be broader than that proposed by Abín et al. (2020), whose results suggested that mathematics anxiety is related to complex but not simple arithmetic, though it must be remembered that Abín et al. (2020) used different measures to ours for both mathematics performance and mathematics anxiety. The meta-analysis by Namkung et al. (2019) also suggested that mathematics anxiety is much more related to complex than simple arithmetic.

As predicted mathematics performance, correlated with working memory; but this only applied to Arithmetic scaled score and not to performance on the written arithmetic test. This may reflect the fact that verbal rehearsal is likely to be more important to oral than written arithmetic (Hitch, 1978b). Also as predicted, Test Anxiety correlated significantly with Mathematics Anxiety/Enjoyment. However, contrary to predictions, it did not correlate with either of with the mathematics measures. It also did not correlate with the working memory measure.

Contrary to predictions, neither working memory, test anxiety nor Mathematics Anxiety/Enjoyment was a significant independent predictor of either of the mathematics measures in multiple regressions. On the other hand, both working memory and test anxiety, but neither of the mathematics performance measures, were significant predictors of Mathematics Anxiety/Enjoyment.

Thus, the mathematics performance measures seemed to be relatively independent of working memory (though this did correlate with Arithmetic scaled score), test anxiety, and even of one another. This differs somewhat from the findings of some other studies, which found stronger influences on mathematical performance of test anxiety (Devine et al., 2012) of working memory (Skagerlund et al., 2019; Caviola et al., 2021) and especially of mathematics anxiety (Ma and Kishor, 1997; Dulaney et al., 2017; Skagerlund et al., 2019; Zhang et al., 2019; Barroso et al., 2021; Caviola et al., 2021). The findings here probably correspond most to those of Douglas and LeFevre (2018), who found relatively limited influence of working memory on other factors and their interrelationships. However, Mathematics Anxiety/Enjoyment did, as pointed out earlier, *correlat*e with all the other variables, even though it did not *independently* predict and was not *independently* predicted by most of them, and it was independently predicted by both working memory and test anxiety. The fact that working memory was a significant independent predictor of attitudes to mathematics, to a greater extent than actual mathematical performance, is one of the most striking findings of the present study. Future research should investigate the direction of causation. It has tended to be assumed that mathematics anxiety interferes with working memory, but it is also possible that working memory weaknesses contribute to mathematics anxiety by increasing the frequency of instances of distraction, confusion, and private and public failures.

As predicted, the ANOVA showed that females and males did not differ in measures of mathematics performance, but females showed more negative attitudes to mathematics, as well as higher levels of test anxiety. These findings are consistent with numerous previous findings, for example, Devine et al. (2012). The lack of a gender difference in performance supports ideas that differences in mathematics anxiety are not explainable by actual poorer performance and may result from exposure to gender stereotypes. They may also reflect differences in academic performance anxiety more generally, a possibility supported by the finding that gender differences in mathematics anxiety ceased to be significant when test anxiety was introduced as a covariate. Future studies should investigate differences in attitudes to and anxiety about academic subjects other than mathematics, including subjects, such as English, where females are generally regarded as higher performing than males. It may also be that different science subjects may be associated with different levels of mathematics anxiety and enjoyment. Some sciences are known to be predominantly chosen by female students (e.g., biology and psychology) and others to be predominantly chosen by male students (e.g., physics and engineering), and it is possible that the latter are seen as “more mathematical.”

The study partially supported the hypothesis that science students would perform better at mathematics than humanities students; they scored higher on the written mathematics test than humanities students, but the two groups of students did not differ in Arithmetic scaled score. Science students reported more positive attitudes to mathematics than did humanities students. This finding is unsurprising because, not only are high level mathematical skills required for entry onto most science degree courses, but those who enjoy mathematics are more likely to select such courses. However, the difference in attitudes was very striking, especially as all the participants, as students at a highly selective university, would have needed to have good mathematics qualifications at 16+, and therefore, people with strongly negative attitudes would have been less likely to be participants in the first place. Unlike the gender difference, this difference applied to mathematics anxiety only, and not to general test anxiety. It is also notable that, though there was no significant course–gender interaction for mean mathematics anxiety scores, male science students appeared to be more homogeneous in their (lack of) mathematics anxiety than the other groups, showing a very low standard deviation.

One of the most striking findings was that science students had longer digit spans, implying better working memory, than the humanities students. It would be of interest to investigate whether this is the case for all sciences or just for some and whether humanities students might do better on tests of long-term memory, as their subjects may involve less need for keeping track of ongoing experimental results and more need to remember information long-term. The present findings are consistent with those of Popescu et al. (2019), who found that mathematics graduate students scored higher on backward digit span and on another working memory task (forward letter span) than humanities graduate students.

Despite the differences in working memory between people doing science and humanities courses, working memory was not driving the differences between courses, as it was not a significant covariate in the ANOVA comparing students taking different courses on attitude and performance measures; and the course differences in written arithmetic and Mathematics Enjoyment/Anxiety were not affected by its inclusion as a covariate.

The most significant limitation to the present study is of course the relatively small sample of 40 participants. Most findings were either clearly significant or non-significant, and there were few of the borderline and near-significant results that can result from underpowering, with the exception of the 0.3 correlation (*p* = 0.06) between Arithmetic Scaled Score and Backward Digit Span, However, it is still possible that some potentially significant associations were not found due to the relatively small sample size and that this may partially explain the limited number of independent predictors found. Future studies should attempt to replicate the findings with larger sample.

Ideally, such a sample should also be more diverse. As is commonly true of studies of adults, the results may be to some degree biased by the fact that the available participants were university students. Thus, it is likely that they were more able mathematically and had more positive attitudes than the general population. For example, no participant obtained an Arithmetic scaled score lower than 10, which represents average performance. It would be desirable to study relationships between mathematics attitudes, mathematics performance, and working memory in a larger and more varied, less self-selected sample; though in any sample, people with severe levels of mathematics anxiety are more likely to decline to participate. The participants in this study were informed in advance about what the study would involve, including a mathematics task. This was deemed necessary for ethical reasons, as important for obtaining informed consent; but it may have deterred people with high levels of mathematics anxiety.

It would also be desirable for future studies to include measures of motivation, which may help to explore the possibility that there is not always a simple linear negative relationship between mathematics performance and mathematics anxiety. Macher et al. (2015) have suggested that anxiety may not always be associated with poor performance, at least in the case of statistics anxiety. They proposed that statistics anxiety may on the one hand, both disrupt performance on the other hand may increase motivation to avoid failure, leading for example to greater preparation for examinations. Wang et al. (2015) found that the relationship between mathematics anxiety and mathematics performance could vary with students’ level of intrinsic motivation toward mathematics. Students with low intrinsic motivation showed a negative relationship between mathematics anxiety and performance. Students with high intrinsic motivation showed an inverted U-shaped relationship between mathematics anxiety and performance: performing best when moderately anxious and least well when either highly anxious or showing very little anxiety.

In any case, it seems that the relationship between mathematics anxiety and mathematical performance is not always simple, especially if motivation is included as a variable. Wang et al. (2018) carried out a further study of over 900 high school students, including profile analysis of combinations of dimensions of mathematics anxiety and mathematics motivation. They found eight different profiles, with different types of associations with mathematics achievement and engagement. For instance, the highest achieving students reported modest examination-related mathematics anxiety and high mathematics motivation, while the most engaged students reported both high examination-related mathematics anxiety and high mathematics motivation.

As stated in the Introduction, the use of Aiken (1974) Mathematics Enjoyment Scale had the advantages of taking relatively little time and of measuring positive as well as purely negative reactions to mathematics, which reduced the chance of ceiling effects in a relatively mathematically able population. However, one problem with the use of a single scale to measure attitudes and emotions toward mathematics is that it makes it more difficult to differentiate between the effects on mathematical performance, and interactions with working memory, of different attitudes and emotions regarding mathematics. In the present paper, “attitudes to mathematics,” “mathematics anxiety,” and “mathematics enjoyment/anxiety” have been used almost interchangeably to refer to negative reactions to mathematics. The use of more diverse measures might facilitate a more nuanced analysis. Since some other studies have suggested that positive emotions toward mathematics predict performance, even after controlling for anxiety (Pinxten et al., 2014; Villavicencio and Bernardo, 2016), it would be interesting to investigate whether different aspects of attitudes and emotions regarding mathematics have different effects on mathematics performance This could involve having separate scales for mathematics anxiety and mathematics enjoyment, and/or incorporating and investigating a range of components of mathematics anxiety and other attitudes and emotions, as is done to varying degrees in, for example, the Mathematics Anxiety Rating Scale (Richardson and Suinn, 1972) and the Fennema-Sherman mathematics attitude scales (Fennema and Sherman, 1976).

There is also the question of how closely attitudes and emotional reactions to mathematics are related, and which of these has the strongest relationship to mathematical performance. Some researchers have suggested that mathematics anxiety has both a cognitive dimension (performance anxiety) and an affective dimension (fearful emotional reactions to mathematical stimuli; Wigfield and Meece, 1988; Sorvo et al., 2017). Some studies suggest that the cognitive dimension is not strongly related to mathematical performance before secondary school age, while the affective dimension is already significantly related to performance in the primary school years (Sorvo et al., 2017). Interestingly, Chen et al. (2018) found that, in a group of elementary school children, attitudes to mathematics were not associated with affective-motivational brain areas, which may indicate that, at least in the early stages of development, attitudes to mathematics are distinct from emotions. It may also indicate that, as several studies have suggested, younger children have more positive attitudes to mathematics than older children and adults and have relatively low levels of mathematics anxiety (e.g., Hembree, 1990; Dowker et al., 2012; Szczygieł and Pieronkiewicz, 2021). Chen et al. (2018) found that in elementary school children, the positive attitudes were associated with enhanced hippocampal activation. They proposed that positive attitudes might influence memory processes in mathematics. Therefore, they suggested that attitudes might influence memory processes during learning activities and task solving.

Some studies have suggested that self-rating may be a stronger predictor of mathematical performance than either anxiety or enjoyment in both primary and secondary school children (Dowker et al., 2012; Van der Beek et al., 2017). It can be difficult to determine the causal direction: part of the relationship could be because individuals are in fact estimating their performance accurately and may for example be rating their performance on the basis of previous test scores. However, even in longitudinal studies, confidence seems to predict future performance (Pinxten et al., 2014). Therefore, future studies should include measure of confidence/self-rating in mathematics.

Therefore, it would be useful to replicate the current study in school children, preferably longitudinally and starting in primary school, to see how the relationships between these variables change over time, as suggested by Vukovic et al. (2013). An important aim would be to see if there is a particular point in childhood where the relationship between mathematics anxiety and mathematics performance typically begin to show a strong correlation. If so, it would be desirable to intervene in either mathematics performance or mathematics anxiety or both, before the development of a vicious circle, which may be hard to break; and, if possible, to create a virtuous circle instead. For example (Supekar et al., 2015) found that a mathematics intervention for young children not only improved mathematical performance but reduced anxiety. There are still fewer interventions for mathematics anxiety than for mathematical performance, and it is important to do more work on developing them (Moustafa et al., 2021). It may be that one next step would be to develop interventions simultaneously targeting both performance and anxiety.

To summarize: the results of the present study suggest that mathematics anxiety is correlated with both simple oral arithmetic and complex written arithmetic, but that it ceases to be a significant predictor of either type of arithmetic when test anxiety and working memory are included with it in a multiple regression. However, test anxiety was neither a significant correlate nor significant independent predictor of either mathematics measure. Working memory was a significant independent predictor of oral but not written arithmetic. In multiple regressions with mathematics anxiety as the dependent variable, it was significantly predicted by both working memory and test anxiety, but not by either measure of mathematical performance. There were no gender differences in oral or written arithmetic or in working memory, but females showed more test anxiety and more mathematics anxiety. There were signs that test anxiety was driving the gender differences in mathematics anxiety, as gender differences in mathematics anxiety ceased to be significant when test anxiety was included as a covariate. As regards course, science students had lower mathematics anxiety than humanities students, but the groups did not differ in test anxiety, Science students were better at written but not mental arithmetic. They were also better at working memory, but this was not a significant covariate and did not appear to be influencing the group differences in written mathematics and mathematics anxiety.

## Data Availability Statement

The raw data supporting the conclusions of this article will be made available by the authors, without undue reservation.

## Ethics Statement

The studies involving human participants were reviewed and approved by Central University Research Ethics Committee, Oxford University. The patients/participants provided their written informed consent to participate in this study.

## Author Contributions

AD was predominantly responsible for designing the study and HS for carrying out the experiments. All authors contributed equally to analyses and wrote the article.

## Funding

This research was partly based on a student project, which was funded by the Oxford University Department of Experimental Psychology.

## Conflict of Interest

The authors declare that the research was conducted in the absence of any commercial or financial relationships that could be construed as a potential conflict of interest.

## Publisher’s Note

All claims expressed in this article are solely those of the authors and do not necessarily represent those of their affiliated organizations, or those of the publisher, the editors and the reviewers. Any product that may be evaluated in this article, or claim that may be made by its manufacturer, is not guaranteed or endorsed by the publisher.
